# Review of Non-Respiratory, Non-Cancer Physical Health Conditions from Exposure to the World Trade Center Disaster

**DOI:** 10.3390/ijerph15020253

**Published:** 2018-02-03

**Authors:** Lisa M. Gargano, Kimberly Mantilla, Monique Fairclough, Shengchao Yu, Robert M. Brackbill

**Affiliations:** New York City Department of Health and Mental Hygiene, Division of Epidemiology, World Trade Center Health Registry, 125 Worth Street, New York, NY 10013, USA; kmantilla@health.nyc.gov (K.M.); mfairclo@health.nyc.gov (M.F.); syu@health.nyc.gov (S.Y.); rbrackbi@health.nyc.gov (R.M.B.)

**Keywords:** 11 September, 2001, rescue and recovery workers, cohort, physical health

## Abstract

After the World Trade Center attacks on 11 September 2001 (9/11), multiple cohorts were developed to monitor the health outcomes of exposure. Respiratory and cancer effects have been covered at length. This current study sought to review the literature on other physical conditions associated with 9/11-exposure. Researchers searched seven databases for literature published in English from 2002 to October 2017, coded, and included articles for health condition outcome, population, 9/11-exposures, and comorbidity. Of the 322 titles and abstracts screened, 30 studies met inclusion criteria, and of these, 28 were from three cohorts: the World Trade Center Health Registry, Fire Department of New York, and World Trade Center Health Consortium. Most studies focused on rescue and recovery workers. While many of the findings were consistent across different populations and supported by objective measures, some of the less studied conditions need additional research to substantiate current findings. In the 16 years after 9/11, longitudinal cohorts have been essential in investigating the health consequences of 9/11-exposure. Longitudinal studies will be vital in furthering our understanding of these emerging conditions, as well as treatment effectiveness.

## 1. Introduction

The collapse of the Twin Towers at the World Trade Center (WTC) on 11 September 2001 after the terrorist attack, which immediately killed nearly 2800 people, provoked multifaceted direct and indirect consequences. As a consequence of structural damage and ensuing fires caused by the total collapse of the towers, an unmeasured amount of toxic and irritant debris was dispersed, resulting in human and environmental repercussions of an enormous magnitude [1]. The crash of the two aircrafts with almost 100,000 liters of jet fuel into the two towers resulted in explosions and extremely high-temperature fires that burned structural elements of the towers as well as their contents [2]. The collapse resulted in the pulverization of as much as one million tons of building materials, including glass, steel, concrete, and sheetrock. The ensuing large plume of smoke released gases and particles in Lower Manhattan and outlying areas [3]. Additional environmental exposures in the immediate aftermath of the disaster also include asbestos, polycyclic aromatic hydrocarbons (PAHs), and polychlorinated furans and dioxins [4,5]. 

Environmental exposures affected workers located at the site of the collapse, community residents, firefighters, police first-responders, iron-workers, and clean-up and recovery workers at the WTC site, including those involved in the restoration of essential services and infrastructures [6]. While most agree that the population at highest risk remains “the Ground Zero” first responders and workers (who were caught in the dust cloud that resulted from the collapse of the WTC towers as well as having chronic exposure while conducting clean-up and recovery work), many survivors, including residents, WTC Tower evacuees, passers-by, and area workers, were exposed to the dust and debris cloud on 9/11. In addition, some were exposed to the toxic debris emanating from the site fires that burned for months after the event. Subsequently, these individuals presented symptoms due to irritating effects of these toxins. In particular, dust particles were highly alkaline and corrosive, thus capable of causing chemical irritation of the eyes and of the upper airway tract [1]. 

While most studies and previous reviews of the physical health effects of 9/11 have generally focused on respiratory diseases and cancer, there is a growing body of evidence that other physical health conditions are associated with various 9/11-exposures. This review explores “emerging” physical health effects of 9/11 other than respiratory effects and cancer to examine what has been learned and to identify areas in which further research is needed. 

## 2. Methods

### 2.1. Search Strategy

Two researchers searched PubMed, CINAIL, Science Citation Index, Google Scholar, EMBASE, PsychLit, and the Cochrane Library for literature published in English from 2002 to October 2017. The search terms used in each database were the following: ((World Trade Center OR 11 September Terrorist Attacks OR WTC OR 11 September 2001) AND (GERD OR autoimmune OR diabetes OR skin OR sarcoidosis OR cardiovascular)). Reference lists of identified articles were also examined in order to identify relevant work that may not have surfaced in the database search. Finally, published reviews addressing WTC conditions were examined to identify additional relevant publications.

### 2.2. Inclusion Criteria

Selection criteria included: (1) full-length original research articles; (2) published in English from 2002 to October 2017; (3) collected data from adults directly exposed to the WTC disaster; (4) analyzed selected physical health conditions attributed to 9/11-exposure that were not cancer or respiratory-related as the outcome.

### 2.3. Study Selection

**Figure 1:**
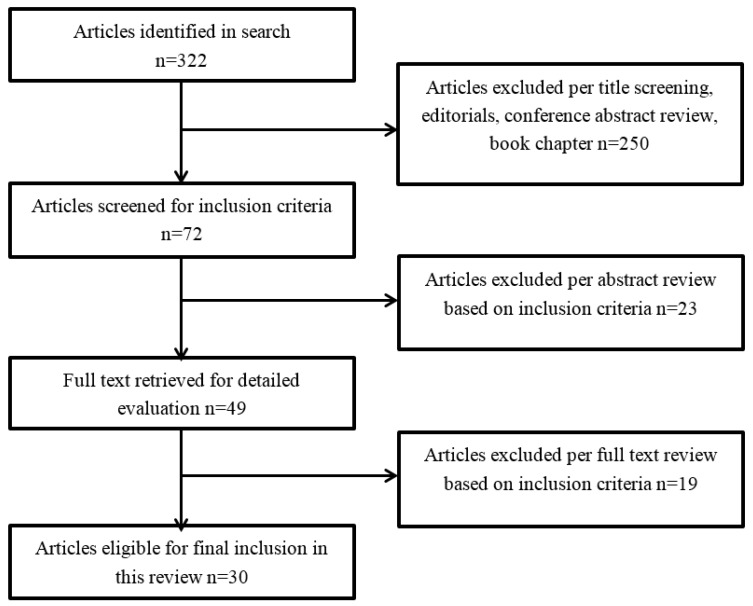
Details of literature review.

All relevant titles, abstracts, and if necessary, papers (*n* = 322) were reviewed independently by two researchers. Articles were excluded if they: (1) were not original research articles (such as reviews, editorials, commentary); (2) collected data only on environmental consequences; (3) collected data only on those indirectly exposed throughout the United States and internationally; (4) collected data exclusively on children under 18 on 9/11; or (5) were government, international organization, and non-governmental organization reports. After the initial screening, 49 articles were selected for inclusion. There was almost perfect agreement between researchers in selecting studies (kappa = 0.90). Following this, a second more detailed screen was done to assess articles more closely. Upon this further screening, 19 articles were excluded because they were not relevant to the review. The researchers independently coded all included articles for demographic and methodological information, as well as any comorbidity assessed. There was good agreement between researchers in coding articles (kappa = 0.87), and discrepancies were discussed and resolved by both researchers and a third reviewer (Figure 1).

## 3. Results

Basic study information is presented in Table 1. In total, 30 studies met inclusion criteria. The study descriptions were categorized into 7 groups, although some covered multiple health conditions, (1) cardiovascular diseases (CVD) (*n* = 9); (2) autoimmune diseases (*n* = 5); (3) Gastroesophageal reflux disease/Gastrointestinal conditions (GERDS/GI) conditions (*n* = 11); (4) obstructive sleep apnea (OSA) (*n* = 2); (5) skin conditions (*n* = 3); (6) Rhinosinusitis (*n* = 4); and (7) “other” which included type II diabetes, hearing loss, and headaches (*n* = 5). Of the included studies, 19 were on rescue and recovery workers (RRW), including those from the Fire Department of New York City (FDNY), New York City Police Department (NYPD), New York City Department of Sanitation (DSNY), Metropolitan Transit Authority (MTA), and volunteers, 2 were on area workers and residences, and 9 were a mix of RRW and non-RRW. The majority of the studies (*n* = 28) came from three cohorts, the World Trade Center Health Registry (WTCHR), the World Trade Center Health Consortium (WTCHC), and the Fire Department of New York (FDNY). Of these, only the WTCHR includes non-RRW. The WTCHC includes several clinic sites, the Stony Brook University World Trade Center General Responders Cohort and Mt. Sinai’s World Trade Center Medical Monitoring and Treatment Program. Table 2 presents the studies and what 9/11-exposures were associated with the health outcome and whether comorbidities were examined.

### 3.1. Cardiovascular/Cerebrovascular Diseases

Cardiovascular diseases (CVD) generally refer to conditions that involve narrowed or blocked blood vessels that can lead to a heart attack, chest pain (angina), or stroke [7]. Well-known risk factors for CVD include smoking, excessive blood lipids, and hypertension, all of which are amenable to effective interventions. However, many studies have also demonstrated a relationship between exposure to ambient air pollutants and CVD [8]. Particulate matter (PM) is generated by combustion of organic matter, which also produces gaseous pollutants. Exposure to PM is associated with adverse health effects leading to increased morbidity [9]. In the case of 9/11-exposed rescue and recovery workers (RRW), such exposures include immersion in the dust cloud and working on the pile at Ground Zero. Particles less than 2.5 μm (PM_2.5_) have been linked with cardiovascular disease [9]. Different components of PM may affect the risk for cardiovascular disease by different mechanisms including electro-physiologic changes, inflammation, coagulation, endothelial cell function effects and increased atherogenesis [10]. 

Three of the eight papers on CVD and 9/11-exposure focused on RRW. Using dynamic contrast-enhanced magnetic resonance imaging, Mani et al. (2013) found that Ground Zero workers with high exposure to particulate matter (exposure to the dust cloud on 9/11) had evidence of more plaque neovascularization, a clinical indicator of atherosclerotic burden, compared to those with low exposure (after 13 September 2001) [10]. The second study, by Moline et al. (2016), compared metabolic syndrome (MetS) of 9/11-exposed law enforcement officers to a national sample. MetS predisposes an individual to have an increased risk of CVD. This study found that the prevalence of MetS was lower, 27%, compared to 45% of a national sample from the National Health and Nutrition Examination Survey (NHANES). Authors attribute this difference to NHANES including working and non-working adults over the age of 20 years, while the Moline study only included only working adults [11]. This study also found that MetS, as well as its risk factors, were significantly higher among male officers compared to female officers. The third study from Perritt et al. (2011) was a descriptive analysis of data collected July 2002 through April 2004 among RRW enrolled in the WTC Health Program which found that 8% (*n* = 375) reported chest pain in this time period [12]. 

Two studies reported increased risks for CVD hospitalization. Jordan et al. (2013) found elevated risks of CVD hospitalization in both male and female RRW with high levels of overall exposure (male adjusted hazard ratio AHR = 1.82, 95% confidence interval 1.06–3.13; female AHR = 3.29, 95% CI 0.85–12.69); hazard ratios for highly exposed non-RRW were not significantly elevated. Post-traumatic stress disorder (PTSD) increased the risk of cerebrovascular disease hospitalization in men but not in women [13]. The presence of PTSD at study enrollment was associated with an elevated risk of subsequent heart disease hospitalizations in women. A high overall level of World Trade Center rescue and recovery–related exposure was associated with an elevated heart disease hospitalization risk in men (AHR 1.82, 95% CI 1.06–3.13; *p* for trend = 0.05), but findings in women were inconclusive (AHR 3.29, 95% CI 0.85–12.69; *p* for trend = 0.09) [13]. A study by Lin et al. (2010) found a significant increase in the rate of cardiovascular disease admissions among residents of lower Manhattan relative to residents of Queens for the weeks of 18 September 2001and 9 October 2001 and for cerebrovascular disease admissions during the weeks of 11 September 2001, 18 September 2001, 2 October 2001, and 8 October 2001 compared to the mean rate during the preceding 10 years. The highest number of cardiovascular admissions coincided with the week of 18 September 2001 and for cerebrovascular admissions, the week of 2 October 2001, demonstrating a delayed increase for cardiovascular admissions compared to those observed for respiratory admissions [14]. 

One study that focused on survivors who were in one of 38 collapsed or damaged buildings by Brackbill et al. (2014) found that compared to those in damaged buildings, WTCHR enrollees who escaped from collapsed buildings had a 30% increased odds of newly diagnosed hypertension [15]. 

Three of the studies included non-RRW. Brackbill et al. (2014) found that persons with multiple injuries and PTSD had a 3-fold higher risk of heart disease than those with no injury and no PTSD [16]. Another study by Jordan et al. (2011) from the WTCHR found that in women, intense dust cloud exposure and injury on 9/11 were significantly associated with heart disease [17]. Injury on 9/11 was also significantly associated with heart disease in men. Participants with PTSD had an elevated heart disease risk for both men and women. In addition, a dose–response relationship was observed between PTSD checklist (PCL) score and heart disease risk [17]. A recent study by Alper et al. (2017) found a dose-response association with the number of injuries sustained on 9/11 and heart disease [18]. 

Two studies also looked at stroke (cerebrovascular disease). One study by Brackbill et al. (2006) found that survivors who were in one of 38 collapsed or damaged buildings found that those who reported being exposed to the dust and debris cloud had more than 5 times increased odds of stroke [15]. The other study, by Jordan et al. (2013) found that PTSD increased the cerebrovascular disease hospitalization risk in men but not in women [13].

### 3.2. Autoimmune Diseases

A growing body of research shows that factors that contribute to the development of autoimmune diseases extend beyond genetics to include environmental/occupational exposures and PTSD. One well-studied environmental exposure is silica, which is commonly used in construction materials, including cement and glass, and was found to be present in the dust cloud on 9/11 [5], and has been associated with several types of autoimmune diseases, including rheumatoid arthritis (RA) [19], systemic sclerosis [20,21], systemic lupus erythematosus [22], dermatomyositis [23], Sjogren’s syndrome [22], and risk of antineutrophil cytoplasmic antibody-associated vasculitis [24]. 

In addition to the exposure to environmental risk factors for autoimmune diseases, PTSD is associated with a number of biological abnormalities that could increase the risk for autoimmune diseases [25,26,27,28,29]. A study of veterans of Iraq and Afghanistan found that those with PTSD were more likely to have autoimmune disorders such as rheumatoid arthritis, multiple sclerosis, lupus, inflammation of the thyroid, and inflammatory bowel disease [30].

Four studies in RRW populations have investigated autoimmune diseases in relation to 9/11-exposures. A study by Loupasakis et al. (2015) found that out of 13,468 FDNY firefighters, 60 developed sarcoidosis, of these, 11 also had polyarticular arthritis [31]. All 11 arrived at the WTC site within the first 3 days after the attack and collapse of the WTC towers, 7 arriving on day 1 [31]. Webber et al. (2015) conducted a nested case-control study among firefighters who had received a rheumatologist-confirmed systemic autoimmune disease diagnosis between 12 September 2001 and 11 September 2013. This study found that odds for acquiring an autoimmune disease increased by 13% for each additional month worked at the WTC site [32]. Another paper by Webber et al. (2015) found that rates of selected systematic autoimmune diseases (RA, lupus, polymyositis/dermatomyositis, psoriatic arthritis, and scleroderma) were not significantly different from expected rates among firefighters. However, the lower 9/11-exposure group, defined as those arriving after 9/11 and a duration of ≤6 months, had 9.9 fewer cases than expected, whereas the higher 9/11-exposure group, defined as those arriving on 9/11 and a duration of ≥7 months, had 7.7 excess cases [33]. Another study by Webber et al. (2016) among 14,966 FDNY and EMT workers found 68 post-9/11 cases of sarcoidosis [34]. Sarcoidosis is the growth of tiny collections of inflammatory cells in different parts of the body [35]. Although the cause of sarcoidosis is unknown, a leading hypothesis is that certain environmental exposures interact with genetic factors to trigger an inflammatory response in susceptible individuals [36]. Overall, FDNY/EMT rates were significantly higher than expected compared to rates from unexposed, demographically similar men in the Rochester Epidemiology Project [34]. In addition, the standardized incidence ratios (SIR) ranged from 2.7 (95% CI 2.0–3.5) in the lower WTC exposure group to 4.2 (95% CI 1.9–8.0) in the most highly exposed [34]. 

There was one study on sarcoidosis among WTCHR enrollees which had a mix of RRW and non-RRW by Jordan et al. (2011). Forty-three cases of post-9/11 sarcoidosis were identified, with 28 incident cases. This case-control study found that working on the WTC pile was associated with sarcoidosis but WTC dust cloud exposure was not [37].

### 3.3. Gastroesophageal Reflux Disease/Gastrointestinal Conditions

Gastroesophageal reflux disease (GERD) is a chronic digestive disorder that occurs when stomach acid or stomach contents flow back into the esophagus causing a heartburn sensation. It is also called acid reflux [38] and has been associated with a poorer quality of life [38,39,40]. Alkaline cement dust, one of the major constituents of WTC dust, has been associated in occupational studies with reflux-like dyspepsia but the biological mechanism is not clear [41]. Barrett’s esophagus, when tissue in the esophagus is replaced by tissue similar to the intestinal lining, is often diagnosed in people with long-term GERD. Barrett’s esophagus is associated with an increased risk of developing esophageal cancer [42]. Several studies have found an association between 9/11-exposure and GERD among both WTC RRW and survivors. The ingestion of alkaline materials in the dust cloud [4] has been a suggested cause of new or worsening GERD in 9/11-exposed firefighters [43]. Furthermore, it has been observed that GERD persists among those RRW who developed this condition after their 9/11-exposures [44,45]. 

Eight studies examined GERD and 9/11-exposure among RRW, and found differences by exposure, comorbidity and demographic variables. A study by Webber et al. (2009) found that symptom report of GERD remained stable between two time points (2001–2002) and (2004–2005) [46]. This study also found that report of lower respiratory symptoms or rhinosinusitis at the first time point increased the odds of GERD at the second time point. In addition, earlier arrival time on 9/11 and months worked at WTC site were associated with GERD, with each additional month of work increasing the odds of symptoms by 8–14%. A study by Wisnivesky et al. (2011), of 27,449 RRW reported that the pre-9/11 cumulative incidence of GERD was 5.8%, this increased to 10.7% at one year post-9/11, 25.4% five years after, and 39.3% nine years after 9/11. Comorbidity was also reported with 42.7% of GERD reported with at least one mental health disorder. The risk of GERD was greatest in workers with the highest levels of exposure (worked more than 90 days, who were caught in the dust cloud, and who worked on the pile at Ground Zero) [45]. Another study of non-FDNY RRW by Perritt et al. (2011) that were part of a medical screening program found that 3% (*n* = 163) reported digestive system illness [12]. Obesity is a risk factor for GERD [47,48]. Findings from a study by Icitovic et al. (2016) of 19,819 RRW were consistent with other research, showing that GERD was associated with 9/11-exposure; however, they further showed that GERD was associated with 9/11-exposure independent of BMI [49]. A study by Yip et al. (2016) of EMT workers found that those who arrived earliest on 9/11 had the highest overall post-9/11 cumulative incidence of GERD [50]. In addition, 22.3% were comorbid for at least one mental health condition and GERD [50]. Jiang et al. (2016), examining gender differences among RRW, found that women were 41% more likely to have GERD than men over the period from 9/11 until the end of 2015 [51]. Women are 85% more likely to develop comorbid asthma and GERD than men, which the authors postulate may be because women are more likely to report symptoms than men [51]. A study by Liu et al. (2017) among 9/11-exposed FDNY found that an obstructive airway disease (OAD) diagnosis significantly increased the risk for subsequent GERD diagnosis. Furthermore, 21% of the WTC exposure effect (high vs. low intensity) on GERD was mediated by a prior OAD diagnosis [52]. Litcher-Kelly et al. (2014) found that psychological distress, including depression, PTSD, panic, and anxiety, reported 3 to 4 years after 9/11 increased the risk of reporting new-onset upper GI symptoms, approximately 6 years after 9/11, in both police and nontraditional WTC responders [53].

Three additional studies on GERD did not focus exclusively on RRW. These studies were all from the WTCHR which used symptom descriptions rather than medical diagnosis, so the condition is referred to as gastroesophageal reflux symptoms (GERS). A study focusing on survivors of the 38 collapsed or damaged buildings by Brackbill et al. (2006) found those exposed to the dust cloud were 1.7 times more likely (AOR: 1.7; 95% CI 1.5–1.9) to report heartburn, indigestion, or acid reflux compared to those who were not in the dust cloud [15]. A study by Li et al. (2011) of adult enrollees in the WTCHR, found the cumulative incidence of GERS was 20% for post-9 /11 GERS and 13% for persistent GERS [44]. Persistent GERS occurred more often among those with comorbid PTSD (24%), asthma (13%), or both (36%) compared with neither of the comorbid conditions (8%). Among enrollees with neither asthma nor PTSD, the adjusted risk ratio for persistent GERS was elevated among workers arriving at the WTC pile on 9/11 or working at the WTC site more than 90 days; residents exposed to the intense dust cloud on 9/11, or who did not evacuate their homes; and area workers exposed to the intense dust cloud. Also noted, there was a dose-response gradient observed in both RRW and non-RRW between the level of 9/11-exposure and GERS within four comorbid strata [44]. A follow-up study, also conducted by Li et al. (2016), investigated the relationship between PTSD, asthma, and GERS more closely. Using mediation analysis, for both RRW and community members, findings showed that asthma diagnosed soon after 9/11 increased the risk of late-onset GERS directly as well as indirectly via an increased PCL score [54].

### 3.4. Obstructive Sleep Apnea 

Obstructive sleep apnea (OSA) is a chronic condition characterized by recurrent episodes of partial or complete upper airway collapse during sleep. Two papers were found to assess whether OSA was associated with 9/11-exposure, both of which focused on RRW. A study by Webber et al. (2011) among FDNY utilized self-administered health questionnaires that consisted of questions about sleep problems. This study found that independent predictors of high risk for OSA included earlier arrival at the WTC site and several 9/11-related conditions such as GERD, chronic rhinosinusitis, and PTSD [55]. The other study by Glaser et al. (2011) utilized polysomnography as an objective measure of OSA. Over 80% (*n* = 514) of participants had some degree of OSA; of those 51% (*n* = 263) had severe OSA. Severe OSA was associated with being present at Ground Zero on 11 September 2001, GERD, and comorbid GERD/rhinosinusitis [56].

### 3.5. Skin Conditions

Skin rash or irritation has been associated with occupational exposure to materials made from fibrous glass, rock wool or slag wool, cement and concrete [57,58,59]. These materials were found in the dust cloud from the collapse of the WTC towers and in the settled dust of residential and office buildings [60]. It is conceivable that post-9/11 skin rash or irritation may be linked to 9/11-exposure. Brackbill et al. (2006) found that building survivors who were caught in the dust cloud were 70% more likely to report skin rash or irritation than those not caught in the dust cloud [15]. A follow-up study of WTCHR enrollees by Huang et al. (2012) found that among 42,025 participants, 12% reported post-9/11 skin rash on an initial survey (2003–2004), 16% on a follow-up survey (2006–2007), and 6% at both times [61]. Initial self-reported post-9/11 skin rash was associated with intense dust cloud exposure, home/workplace damage, and working 31 to 90 days or more than 90 days at the WTC site [61]. A study of RRW and volunteers by Perritt et al. (2011) found that from July 2002 through April 2004, 178 (4%) study participants reported skin rash or irritation [12]. 

### 3.6. Rhinosinusitis

Rhinosinusitis is defined as an inflammation of the mucous membrane that lines the paranasal sinuses and is classified chronologically into several categories, from acute (symptoms usually less than 4 weeks) to chronic (symptoms usually more than 12 weeks) [62]. The dust generated by the collapse of the WTC towers and the diesel exhaust fumes produced by rescue and recovery machinery might have caused inflammation of responders’ airways, which could have led to irritant-induced rhinosinusitis. Four studies examined the association between rhinosinusitis and 9/11-exposure in RRW. One study by Wisnivesky et al. (2011) found that the 9-year cumulative incidence of sinusitis was 42.3% (*n* = 5870) [45]. Incidence was highest in workers with greatest 9/11-exposure. Extensive comorbidity was reported within and between sinusitis, asthma, and GERDS [45]. Another study by Weakley et al. (2015) looked at latency in diagnoses of chronic rhinosinusitis resulting from 9/11-exposure. The effect of exposure to the WTC disaster on the incidence of chronic rhinosinusitis diagnoses persisted for 10 years after initial exposure. The relative incidence by exposure group (high vs. moderate vs. low) of chronic rhinosinusitis disease did not significantly change over the study period. The relative rate of developing chronic rhinosinusitis was 1.99 (95% CI 1.64–2.41) for high versus low exposure, and 1.52 (95% CI 1.28–1.80) for moderate versus low exposure during the 10-year follow-up period [63]. A study by Yip et al. (2016) which focused on FDNY’s NYC Emergency Medical Service (EMS) workers found the 12-year post-9/11 cumulative incidence (11 September 2001 to 31 December 2013) of rhinosinusitis was 10.6% [50]. Compared with unexposed, EMS workers who arrived earliest at the site had a higher adjusted relative risk (aRR) for rhinosinusitis (aRR = 3.7; 95% CI 2.2–6.0) [50]. A study by Liu et al. (2017) among 9/11-exposed FDNY found that an OAD diagnosis significantly increased the risk for subsequent chronic rhinosinusitis diagnosis. Furthermore, 13% of the WTC exposure effect (high vs. low intensity) on chronic rhinosinusitis was mediated by a prior OAD diagnosis [52].

### 3.7. Other Emerging Conditions

Other conditions are starting to emerge in the literature that may be related to 9/11-exposure. Many of these only have one published report but are garnering attention as late-emerging health conditions. These include diabetes [64], headaches [12,15,65], and hearing problems/loss [15]. 

The National Epidemiologic Survey on Alcohol and Related Conditions observed an increased risk of diabetes in those with PTSD [66], which is a concern since PTSD is one of the most common mental health outcomes observed in 9/11-affected populations [67,68]. A study of WTCHR enrollees investigated whether enrollees have an increased risk of diabetes. This study by Miller et al. (2014) found that 2143 (5.8%) enrollees reported having been diagnosed with diabetes between Registry enrollment (2003–2004) and March 2012. While 9/11-exposure was not associated with new-onset type II diabetes, there was a significant association between 9/11-related PTSD and new-onset type II diabetes. In addition, residents of lower Manhattan on 9/11 were found to have lower odds of reporting new-onset type II diabetes compared to RRW [64].

Survivors, rescue-recovery and cleanup workers and NYC transit workers, all of whom were exposed to pulverized steel, glass, cement and other debris in the dust cloud, have experienced headaches years after 9/11. In a study by Tapp et al. (2005) of 269 NYC transit workers who were working near the World Trade Center, 50% reported headaches [65]. In another analysis by Brackbill et al. (2006), of the 1753 building survivors caught in the dust cloud, 26.2% reported severe headaches [15]. A study of RRW by Perritt et al. (2011) with data collected July 2002 through April 2004 found that 8% (*n* = 359) reported headaches in this time period [12]. The association between the exposure to the dust cloud and headaches is still unclear, and more research needs to be conducted on this correlation.

A study by Brackbill et al. (2006) examined self-reported non-respiratory problems after 9/11 among building survivors enrolled in the WTCHR. This study found that 8.1% (*n* = 673) reported hearing problems or hearing loss. The odds of reporting hearing problems or hearing loss were greater for those who reported being caught in the dust and debris cloud and who were in collapsed buildings [15]. A more recent study by Stein et al. (2017) of hearing loss among RRW in the WTCHR found the prevalence of incident, persistent hearing problems was 4.4% [69]. Workers with higher environmental hazards scores (a cumulative exposure to the dust and debris) were twice as likely (interquartile range OR 2.1; 95% CI 1.8–2.5) to report hearing problems and workers who reported being unable to hear in the dust cloud were 2.3 (95% CI 1.8–3.0) times more likely to report hearing problems as compared with workers not in the dust cloud [69].

## 4. Discussion

This article summarized the literature on non-respiratory, non-cancer physical health outcomes among different persons directly exposed to the attacks on 9/11. Over the past 16 years since the attacks, research on the links between 9/11-exposure and physical health conditions such as GERD, CVD, and rhinosinusitis has grown. Research findings on other conditions, like autoimmune diseases and OSA are beginning to emerge, while other conditions such as headaches and hearing problems are only beginning to be studied. Studies on some of these conditions, such as diabetes, hearing loss, and OSA, at this early stage, may be primarily hypothesis-generating. More rigorous methods such as case-control studies and medical verification may be needed to substantiate these findings. Only GERD and OSA are currently covered by the World Trade Center Health Program [70], but the ongoing research into other emerging conditions will be crucial if other conditions are to be added. 

Investigations into the emerging health effects of the 9/11 attacks have unique strengths, including the presence of several large prospective cohorts. Development of these cohorts after both man-made and natural disasters is key for the monitoring of exposed persons and tracking trends of potential emerging health conditions. The evidence base regarding the association between 9/11-exposure and non-respiratory, non-cancer physical health conditions is in its nascent stage but there are multiple instances of supportive evidence for emerging conditions identified in this review. First, there is a consistency of findings across studies, even when different methods or populations are used, studies from RRW and non-RRW populations had similar findings for CVD, GERD, and headaches. Second, associations between self-reported disease and exposure are supported by the use of objective measures, such as hospital records and clinical assessment, as is seen with CVD. 

Because research into these physical health conditions is still emerging, this review also identified areas in the current research that need to be strengthened. There is a larger body of research on these conditions among RRW than non-RRW, 18 of the 29 papers focus solely on RRW and another seven included RRW in the study population. This is problematic when examining the relationship between exposure and health outcome and trying to generalize findings. First, RRW differ inherently from the civilian population in terms of physical health, work exposures, and preparedness and emergency training. Second, the 9/11-exposures of RRW differed from civilians. Many RRW worked on the pile at Ground Zero, exposing them for longer periods of time to particulate matter and smoke and fumes from the fires [67,68]. Third, there are also more studies on some conditions, like GERS and CVD, compared to others. Fourth, some of these conditions, such as autoimmune diseases, are relatively rare. A similar approach which could be used and which is currently being used to study cancer among 9/11-exposed is the pooling of individual data from each of the cohorts [71]. Fifth, some of these conditions represent symptoms for which it can be hard to define a case, such as skin rash, headaches, and hearing loss compared to more clearly diagnosable conditions, such as heart attacks. Therefore, attention will be needed to ensure that rare conditions are being investigated with some of the same objective measures and across different populations. Finally, while over half of the studies included assessing comorbidities, most of these were not among studies of RRWs. In addition, more studies are needed to assess how these comorbidities affect quality of life, disability, treatment effectiveness, and healthcare seeking behaviors. 

Lessons from existing research can provide guidance for future studies. First, treatment of these emerging disorders related to the 9/11 disaster should be assessed. For example, previous research has shown that 9/11-related asthma is resistant to standard treatment [72,73], so it is important to see how these diseases respond to the traditional course of treatment. In addition, there needs to be a focus on studies of treatment effectiveness and best practices in those with and without comorbidities. Second, more information is needed on the pathophysiology of 9/11-related health conditions. Finally, since these cohorts are longitudinal, information can be collected on subsequent traumas after 9/11 that may affect the progression of health disorders. Thus continued follow-up of 9/11-exposed people is needed to document the course of these illnesses and treatment effectiveness.

## 5. Conclusions 

The literature on the burden of non-respiratory, non-cancer physical health conditions among WTC-exposed populations is growing. Longitudinal studies will help clarify the relation between WTC exposure and new-onset physical health outcomes. By identifying key risk factors for morbidity, epidemiologic studies of WTC-exposed populations can guide treatment efforts and inform future disaster response activities.

## Figures and Tables

**Table 1:** Information about included studies.

Author, Year	Condition	Study Design	Data Source of Condition	Population	Sample Size of Interest
Alper et al., 2017 [18]	Cardiovascular disease	Cohort	Self-report	WTC Health Registry Adult enrollees (18–64 years old)	8701
Brackbill et al., 2014 [16]	Cardiovascular disease	Cohort	Self-report	WTC Health Registry Adult enrollees (18–63 years old)	14,087
Jordan et al., 2011 [17]	Cardiovascular disease	Cohort	Self-report	WTC Health Registry: Residence of lower Manhattan and Rescue and Recovery Workers	39,324
Jordan et al., 2013 [13]	Cardiovascular disease	Cohort	Hospitalization records	WTC Health Registry: Residence of lower Manhattan and Rescue and Recovery Workers who lived in New York State	46,417
Lin et al., 2010 [14]	Cardiovascular disease	Case-Control	Hospitalization records	Affected population in Manhattan living near Ground Zero; Control population in Queens County	Affected population: 56,013 Control population: 88,017
Mani et al., 2012 [10]	Cardiovascular disease	Cohort	Clinical assessment	WTCHC: Rescue and Recovery Workers (Law Enforcement)	31
Moline et al., 2016 [11]	Cardiovascular disease	Cohort	Clinical assessment	WTCHC: Rescue and Recovery Workers (Law Enforcement)	2497
Jordan et al., 2011 [37]	Autoimmune disease (sarcoidosis)	Case-control	Clinical assessment	WTC Health Registry: Residence of lower Manhattan and Rescue and Recovery Workers	Cases: 28 Controls: 109
Loupasakis et al., 2015 [31]	Autoimmune disease (sarcoid arthritis)	Cohort	Clinical assessment	FDNY: Rescue and Recovery Workers	13,468
Webber et al., 2015 [32]	Autoimmune disease (systemic autoimmune diseases)	Case-control	Clinical assessment	FDNY: Rescue and Recovery Workers	Cases: 59 Controls: 236
Webber et al., 2016 [33]	Autoimmune disease (systemic autoimmune diseases)	Case-cohort	Clinical assessment	FDNY: Rescue and Recovery Workers	13,892
Webber et al., 2017 [34]	Autoimmune disease (sarcoidosis)	Case-cohort	Clinical assessment	FDNY: Rescue and Recovery Workers (Firefighters and EMT)	13,098
Li et al., 2011 [44]	GERD/GI	Cohort	Self-report	WTC Health Registry: Residence of lower Manhattan and Rescue and Recovery Workers	37,118
Li et al., 2016 [54]	GERD/GI	Cohort	Self-report	WTC Health Registry: Residence of lower Manhattan and Rescue and Recovery Workers	29,406
Litcher-Kelly et al., 2014 [53]	GERD	Cohort	Clinical assessment	WTCHC: Rescue and Recovery Workers (Law Enforcement and Nontraditional)	10,953
Webber et al., 2009 [46]	GERD/GI	Cohort	Self-report	FDNY: Rescue and Recovery Workers	10,378
Glaser et al., 2014 [56]	Obstructive sleep apnea	Cross-sectional	Clinical assessment	FDNY: Rescue and Recovery Workers	636
Webber et al., 2011 [55]	Obstructive sleep apnea	Cohort	Self-report	FDNY: Rescue and Recovery Workers	13,330
Huang et al., 2012 [61]	Skin rash or irritation	Cohort	Self-report	WTC Health Registry: Residence of lower Manhattan and Rescue and Recovery Workers	42,025
Weakley et al., 2015 [63]	Rhinosinusitis	Cohort	Clinical assessment	FDNY: Rescue and Recovery Workers	9848
Miller-Archie et al., 2014 [64]	Other (diabetes)	Cohort	Self-report	WTC Health Registry: Residence of lower Manhattan and Rescue and Recovery Workers	36,899
Stein et al., 2017 [69]	Other (hearing loss)	Cohort	Self-report	WTC Health Registry: Rescue and Recovery Workers	16,579
Tapp et al., 2005 [65]	Other (headaches)	Cross-sectional	Self-report	NYC Transit Workers	269
Multiple conditions					
Brackbill et al., 2006 [15]	GERD/GI, Other (headaches, hearing problems/loss), Skin rash, Cardiovascular disease	Cohort	Self-report	WTC Health Registry Adult enrollees (survivors of collapsed and damage buildings)	8418
Liu et al., 2017 [52]	Rhinosinusitis; GERD	Cohort	Clinical assessment	FDNY: Rescue and Recovery Workers	8968
Perritt et al., 2011 [12]	Cardiovascular disease; GERD/GI; Skin rash/irritation; Other (headaches)	Cohort	Self-report	WTCHC: Rescue and Recovery Workers and Volunteers	7810
Wisnivesky et al., 2011 [45]	Rhinosinusitis; GERD	Cohort	Self-report	WTCHC: Rescue and Recovery Workers; Construction Workers; Municipal Workers	27,449
Yip et al., 2016 [50]	Rhinosinusitis; GERD	Cohort	Clinical assessment	FDNY: Rescue and Recovery Workers (EMT)	2281

GERD: Gastroesophageal reflux disease; GI: Gastrointestinal; FDNY: New York City Fire Department; WTCHC: World Trade Center Health Consortium.

**Table 2:** Health conditions with associated 9/11-exposures and assessed comorbid conditions.

Author, Year	Condition	9/11-Exposure	Comorbid Conditions Reported
Alper et al., 2017 [18]	Cardiovascular disease	Injury	PTSD
Brackbill et al., 2006 [15]	GERD/GI, Other (headaches, hearing problems/loss), Skin rash, Cardiovascular disease (heart disease, stroke, hypertension)	Dust/debris cloud; building type	None assessed
Brackbill et al., 2014 [16]	Cardiovascular disease	Injury	PTSD
Glaser et al., 2014 [56]	Obstructive sleep apnea	Arrival to Ground Zero on 9/11	GERD and comorbid GERD/Rhinosinusitis
Huang et al., 2012 [61]	Skin rash or irritation	Dust/debris cloud; home/workplace damage; working more than 90 days or 31 to 90 days at the World Trade Center site	None found
Icitovic et al., 2016 [49]	GERD/GI	RRW	Obesity
Jiang et al., 2016 [51]	GERD/GI	RRW	None assessed
Jordan et al., 2011 [37]	Autoimmune disease (sarcoidosis)	Worked on the pile at ground zero on 9/11	None assessed
Jordan et al., 2011 [17]	Cardiovascular disease	Dust cloud (women); injury (men and women)	PTSD
Jordan et al., 2013 [13]	Cardiovascular disease	RRW	PTSD
Li et al., 2011 [44]	GERD/GI	RRW; dust cloud; not evacuating home (residents)	PTSD; asthma; PTSD/asthma comorbidity
Li et al., 2016 [54]	GERD/GI	RRW	PTSD; asthma
Lin et al., 2010 [14]	Cardiovascular disease	Resident proximity to Ground Zero	None assessed
Litcher-Kelly et al., 2014 [53]	GERD	RRW; worked on the pile at ground zero on 9/11	Depression; generalized anxiety; panic; PTSD
Liu et al., 2017 [52]	Rhinosinusitis; GERD	Arrival to Ground Zero on 9/11	OAD
Loupasakis et al., 2015 [31]	Autoimmune disease (sarcoid arthritis)	RRW	None assessed
Mani et al., 2012 [10]	Cardiovascular disease	Arrival to Ground Zero on 9/11	None assessed
Miller-Archie et al., 2014 [64]	Other (diabetes)	Exposure index (Very high/high)	PTSD
Moline et al., 2016 [11]	Cardiovascular disease	RRW	None assessed
Perritt et al., 2011 [12]	Cardiovascular disease; GERD/GI; Skin rash/irritation; Other (headaches)	RRW	None assessed
Stein et al., 2017 [69]	Other (hearing loss)	RRW; dust cloud	PTSD
Tapp et al., 2005 [65]	Other (headaches)	Dust cloud	None assessed
Weakley et al., 2015 [63]	Rhinosinusitis	Earlier time of arrival at the WTC site (morning of 9/11 through 9/12)	None assessed
Webber et al., 2009 [46]	GERD/GI	Arrival on 9/11 and prolonged work at Ground Zero	Lower respiratory symptoms; Rhinosinusitis
Webber et al., 2011 [55]	Obstructive sleep apnea	Earlier time of arrival at the WTC site (morning of 9/11 through 9/12)	GERD; chronic rhinosinusitis; PTSD
Webber et al., 2015 [32]	Autoimmune disease (systemic autoimmune diseases)	Prolonged work at Ground Zero	None assessed
Webber et al., 2016 [33]	Autoimmune disease (systemic autoimmune diseases)	Arrival on 9/11 and/or prolonged work at Ground Zero	None assessed
Webber et al., 2017 [34]	Autoimmune disease (sarcoidosis)	Arrival on 9/11 and/or prolonged work at Ground Zero	None assessed
Wisnivesky et al., 2011 [45]	Rhinosinusitis; GERD	Dust cloud; Prolonged work at Ground Zero	GERD; asthma; Rhinosinusitis
Yip et al., 2016 [50]	Rhinosinusitis; GERD	Earlier arrival at Ground Zero	GERD; Rhinosinusitis; obstructive airway disease; mental health disorders

GERD: Gastroesophageal reflux disease; GI: Gastrointestinal; SPD: Serious Psychological Distress; PTSD: Post-traumatic Stress Disorder; RRW: Rescue and Recovery Work; MDD: Major Depressive Disorder; OAD: Obstructive Airway Disease.
